# A Chatbot-Based Coaching Intervention for Adolescents to Promote Life Skills: Pilot Study

**DOI:** 10.2196/16762

**Published:** 2020-02-14

**Authors:** Silvia Gabrielli, Silvia Rizzi, Sara Carbone, Valeria Donisi

**Affiliations:** 1 eHealth Unit Fondazione Bruno Kessler Trento Italy

**Keywords:** life skills, chatbots, conversational agents, mental health, participatory design, adolescence, bullying, cyberbullying, well-being intervention

## Abstract

**Background:**

Adolescence is a challenging period, where youth face rapid changes as well as increasing socioemotional demands and threats, such as bullying and cyberbullying. Adolescent mental health and well-being can be best supported by providing effective coaching on life skills, such as coping strategies and protective factors. Interventions that take advantage of online coaching by means of chatbots, deployed on Web or mobile technology, may be a novel and more appealing way to support positive mental health for adolescents.

**Objective:**

In this pilot study, we co-designed and conducted a formative evaluation of an online, life skills coaching, chatbot intervention, inspired by the positive technology approach, to promote mental well-being in adolescence.

**Methods:**

We co-designed the first life skills coaching session of the CRI (for girls) and CRIS (for boys) chatbot with 20 secondary school students in a participatory design workshop. We then conducted a formative evaluation of the entire intervention—eight sessions—with a convenience sample of 21 adolescents of both genders (mean age 14.52 years). Participants engaged with the chatbot sessions over 4 weeks and filled in an anonymous user experience questionnaire at the end of each session; responses were based on a 5-point Likert scale.

**Results:**

A majority of the adolescents found the intervention useful (16/21, 76%), easy to use (19/21, 90%), and innovative (17/21, 81%). Most of the participants (15/21, 71%) liked, in particular, the video cartoons provided by the chatbot in the coaching sessions. They also thought that a session should last only 5-10 minutes (14/21, 66%) and said they would recommend the intervention to a friend (20/21, 95%).

**Conclusions:**

We have presented a novel and scalable self-help intervention to deliver life skills coaching to adolescents online that is appealing to this population. This intervention can support the promotion of coping skills and mental well-being among youth.

## Introduction

### Background

Adolescence is a challenging period characterized by rapid changes [1] and increasing emotional and social demands. Interventions that foster life skills, coping, and well-being are particularly important during adolescence; virtual coaching solutions could greatly enhance delivery of these interventions in both school and out-of-school settings. The aim of this study was to co-design a life skills coaching intervention with adolescents to be delivered by a chatbot; this would be done by conducting an initial participatory design workshop followed by testing the feasibility of the whole intervention with another convenience sample of adolescents. The life skills virtual coaching intervention was meant to be used either in out-of-school settings for individual training on life skills or in combination with school interventions on life skills provided by a human coach (ie, teacher or domain expert).

### Literature Review

Several challenges and emotional demands characterize adolescence, often affecting the mental well-being of youths. Among these, bullying and cyberbullying is recognized nowadays as a major social problem, affecting 37% of adolescents [2,3], with extensive negative consequences for the victims involved. Research has shown that adolescent bully victimization is associated with poorer school achievement [4]; lower self-esteem; and increased loneliness, depression, and anxiety [5], whose consequences persist into adulthood [6]. Interventions that foster life skills [7], coping, and well-being are particularly important during adolescence as protective and preventive strategies against the consequences of bullying and cyberbullying. Life skills include the ability to exhibit adaptive and positive behaviors that enable individuals to deal effectively with the demands, challenges, and stress of daily life [8]. Childhood and adolescence are the developmental stages during which one acquires these skills through various methods and people [9]; a positive technology approach [10,11] can support the design of such experiences in a digital format. These solutions have the potential to be highly scalable, since almost three-quarters of adolescents (73%) have a mobile phone or have access to one [12]; as well, digital interventions have been proven to be effective at changing a range of health behaviors [13]. Digital assistants or chatbots are conversational agents that can be easily used to support the delivery of educational interventions for mental health and well-being [14]. A chatbot conducts an interaction through conversation with its users by simulating humans’ dialogue patterns and behaviors. However, there is still a paucity of research showing the design and effective usage of chatbot interventions for supporting mental well-being of adolescents [15].

## Methods

### Phase 1: Co-Design Workshop and Participants

The co-design phase of the study involved a sample of 20 adolescents (age range 14-15 years) attending a first-year class of a secondary school in Northeast Italy. A participatory design workshop was organized and led by a research staff of four psychologists. The staff involved the students in using and commenting upon a prototyped session of the chatbot intervention to collect their needs and preferences on the following: the chatbot’s look and feel, the type of content and duration of the session, their unmet expectations regarding the prototype, and suggested improvements. Participants provided signed parental consent. Discussions held during the initial co-design workshop were audio-recorded and transcribed. Qualitative data collected were analyzed using a modified version of Braun and Clarke’s guidelines [16] for thematic analysis. Specifically, two members of the research team read the transcripts and independently developed categories of responses. Agreement on the proposed categories was reached through discussion.

**Figure 1:**
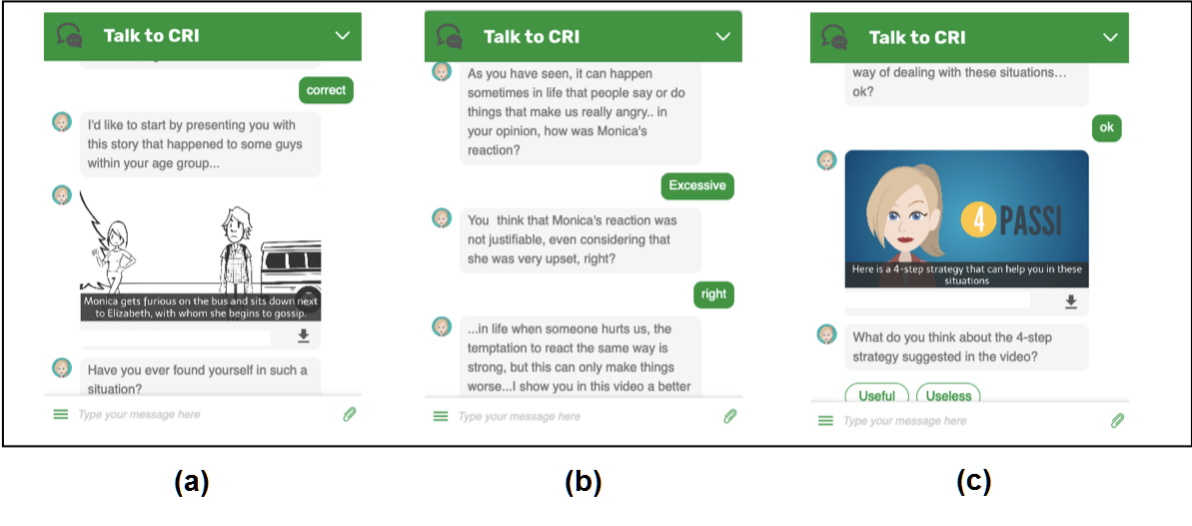
Screenshots of the chatbot conversation with a female user during the coaching session on conflict resolution: (a) initial video on challenging situation, (b) example of coaching questions triggering self-reflection, and (c) final video coaching the user on coping strategies.

Based on the co-design results, the research staff developed the entire life skills coaching intervention, including eight online sessions, delivered by means of a series of coaching dialogues, exercises, and video cartoons presented by the CRI (for girls) and CRIS (for boys) chatbot—the chatbot was named CRI and CRIS as abbreviated forms of the Italian names Cristina (female) and Cristiano (male), respectively. As shown in Figure 1, each session starts by chatting with the CRI or CRIS chatbot, which looks like an older peer—boy or girl—of the user. The chatbot asks the user to watch a 1-2-minute video cartoon about a bullying or cyberbullying situation involving adolescents, which is relevant to the skill addressed, embedded within the conversation system. The chatbot then continues the conversation by asking questions and proposing exercises to trigger user self-reflection on how to cope and behave in such a situation. By the end of the session, the chatbot asks the user to watch another video cartoon where the CRI or CRIS coach provides advice on constructive strategies for coping well with the challenges and emotions triggered by the situation (see Multimedia Appendix 1 for an example of the coaching session). Each session was designed to stimulate self-reflection on life skills and coping strategies relevant to bullying or cyberbullying situations [9], including emotional self-awareness, social awareness, interpersonal relationships, conflict resolution, assertive communication, sadness and loneliness, leadership, and positive emotions. Each session lasted between 5 and 15 minutes. To facilitate engagement, the coaching sessions were personalized according to gender; the chatbot represented a peer of the same gender—girl or boy—as the participant (see Table 1).

**Table 1:** Sample content of the coaching sessions.

Life skill session	Sample of dialogue messages
Emotional self-awareness	... understanding how our feelings and emotions work is the first step to feel better equipped for life and more self-confident ... have you ever thought about this? ... we can learn how to give room to anger, to knowing it, watching it with calm and curiosity as we may watch new things. My hint is to use breathing as a means ...
Social awareness	Take a look at your mobile phone and your messages: which are the emoji you use the most? Which are the ones you receive most often? Let’s exercise by playing a game! I’ll show you an emotion, you guess which one it is by choosing one of three options.
Interpersonal relationships	Our relationships depend very much on the way we give meaning to the characteristics of people we meet. We are generally attracted to people who are similar to us, or to those we would like to be like. Learning to recognize and accept these traits in ourselves or others may help us to overcome prejudice and improve our relationships with others.
Conflict resolution	... in life when someone hurts you, you are tempted to react in the same way, but this can only make things worse ... in this video, I show you a better way of dealing with these situations ... OK? Often interpreting a situation is easier than just observing it in terms of facts ... but this can really help us to not be overwhelmed by negative emotions, like anger and jealousy ... do you agree?
Assertive communication	When you communicate in an assertive manner, your feelings are closely related to the situation you are experiencing at that time and you act constructively. I issue you a challenge: next time you speak with someone, pay attention to the words you are using, to the feelings you experience, to your body language ...
Sadness and loneliness	Sadness and loneliness are basic feelings, but very often we find it hard to express them, because in our society, especially on social networks, most people tend to be happy and smiling ... in short, it looks cooler to be happy ... right? Trying to change our thoughts or do something pleasurable is a good way to feel better ... try to do this in the next few days when you feel sad for any reason ...
Leadership	Although we often think about leaders as popular people, each of us can be a leader, by nurturing the right qualities. Watch this video to better understand the story of Matteo ... Being a good leader is not a trait we need to have from birth, but we can achieve it by being committed and doing our exercises! How? Watch this video ...
Positive emotions	We need to train our brains to see and value positive things. Let's see an example ... Try to write below at least one positive thing that happened to you (even small things, such as “a buddy was kind to me,” “there is sunshine today,” ...). The good news is that we can learn to be happy, by nurturing optimism, openness, and trust!

### Phase 2: Feasibility Test Participants and Setting

A convenience sample of 21 adolescents (13/21, 62% male; 8/21, 38% female) in the age range of 12-17 years (mean 14.52) were invited to use the entire coaching intervention over 4 weeks, participating in two sessions per week. The aims of this formative and qualitative study were as follows: (1) to assess the perceived value of the coaching intervention for a population of adolescents having a wider age range with respect to the group involved in the co-design workshop and (2) to check the user experience with the full set of online coaching sessions in order to refine and finalize their content. Due to the focus of our study—assessing human factors of the virtual coaching experience provided—and the homogeneity of our target group, a sample size of around 20 adolescents was considered appropriate [17,18].

After providing signed parental consent, participants were instructed to access the chatbot from any preferred device by visiting a website where the coaching sessions had been published for the formative evaluation. An anonymous 10-item satisfaction questionnaire was delivered online at the end of each coaching session; a 5-point Likert scale was used to rate overall usefulness, ease of use, and the value of the program, and suggestions for improvements were provided via open-ended questions. Descriptive statistics were calculated for satisfaction items.

## Results

### Phase 1

Regarding the interaction with the prototyped session in the co-design phase, three main themes emerged. First, participants found the educational video cartoons provided in the session to be appealing and helpful in triggering self-reflection on the challenging situation presented: “The black and white video presenting a real-life setting helped me to reflect more on myself, how I would react in that case ...” Second, participants reported that the need to type answers during the chat when replying to the chatbot’s questions helped them to reflect on their feelings and thoughts relevant to the topic, even if they knew that the chatbot was not able to understand their replies as a human being: “Even if I know that the chatbot cannot reply as a real person, since it is not able to understand everything, the fact of typing in the chat what I think about the situation helps me to reflect ...” Third, participants said that the chatbot looked like a nice, smart old friend, someone you can trust and talk with when you want to address important issues: “I like this chatbot, he looks like a boy, just a bit older than me, but not an adult; he is sensitive and very smart.”

With respect to potential improvements, participants found that some parts of the dialogue with the chatbot were a bit unclear or were missing empathy from the chatbot side: “I got confused; after a video, I don’t know what I should say to go on with the session” and “It would be great if the chatbot would reply with something more relevant and empathic to my comment.” Second, some participants reported that parts of the session were too redundant or the chatbot provided too much text that required scrolling up the chat window: “... at some points, the bot gets a bit repetitive, it repeats the same thing many times” and “When the bot sends too much text in a turn, I need to scroll up not to miss the first lines and this is a bit annoying.” Supporting quotes are displayed in Table 2.

**Table 2:** Examples of participant quotes supporting the themes.

Theme	Frequency of quotes	Examples of supporting quotes
Videos support self-reflection	5	I think the video contents were more useful than written sentences. The black and white video presenting a real-life setting helped me to reflect more on myself, how I would react in that case.
Typing answers to reply to the chatbot supports self-reflection	6	I understand that the chatbot doesn’t read what I am typing for real, but this is not a problem because typing makes me think about something and this is important per se. ... even if I know that the chatbot cannot reply as a real person, since it is not able to understand everything, the fact of typing in the chat what I think about the situation helps me to reflect ...
The chatbot looks like a nice, smart, trustworthy old friend	4	I like this chatbot, he looks like a boy, just a bit older than me, but not an adult; he is sensitive and very smart. ... this chatbot is serious, since the things he talks about are important, it makes me think about something I normally do not reflect upon.
Unclear dialogue and chatbot not empathic	5	It's not nice to write something personal ... and then the chatbot goes on without considering it ... it would be nice if he could react in a more empathic way to what I type. I got confused; after a video, I don’t know what I should say to go on with the session. It would be great if the chatbot would reply with something more relevant and empathic to my comment.
Redundancy in some sessions and the chatbot provided too much text that required scrolling up	6	At some points, the bot gets a bit repetitive, it repeats the same thing many times. When the bot sends too much text in a turn, I need to scroll up not to miss the first lines and this is a bit annoying.

### Phase 2

All 21 participants involved in the feasibility test completed the eight coaching sessions and the relevant questionnaires. For all eight sessions, on a scale of 1 (very little) to 5 (very much), the majority of participants (16/21, 76%) gave a rating of 4 or 5 in response to the question “How useful was this session for you?” Ratings were also 4 or 5 for *ease of use* (19/21, 90%) and *innovativeness* (17/21, 81%) of sessions. Participants' ratings on *usefulness* and *ease of use* for each specific session are reported in Table 3. Note that all sessions have a mean rating above 3, corresponding to the positive side of the scale, for both *usefulness* and *ease of use*.

Regarding the design of the contents provided in the sessions, 71% (15/21) of participants reported a preference for the coaching videos and 66% (14/21) expressed a preference for having each session last between 5 and 10 minutes. Among the possible *improvements*, participants mentioned a wider range of answer options to some of the chatbot questions and suggested removing redundancy in the dialogue of the longer sessions. Regarding perceived *value*, 95% (20/21) of the participants indicated they would recommend the CRI or CRIS chatbot to a friend.

**Table 3:** Ratings for *usefulness* and *ease of use* for the eight coaching sessions.

Coaching session	Usefulness, mean (SD)^a^	Ease of use, mean (SD)^a^
Emotional self-awareness	3.60 (0.60)	4.15 (0.45)
Social awareness	3.70 (0.66)	3.92 (0.54)
Interpersonal relationships	4.06 (0.60)	4.53 (0.64)
Conflict resolution	4.33 (0.69)	4.56 (0.71)
Assertive communication	4.14 (0.71)	4.50 (0.75)
Sadness and loneliness	3.89 (0.65)	3.98 (0.66)
Leadership	4.00 (0.66)	4.44 (0.70)
Positive emotions	3.09 (0.53)	4.58 (0.61)

^a^Rating scores range from 1 (very little) to 5 (very much).

## Discussion

### Principal Findings

To our knowledge, today there is still a paucity of chatbot-based interventions for life skills training and well-being promotion among adolescents, as well as a lack of data and guidelines on how to effectively design the user experience with educational chatbots for this intervention domain. However, use of Internet and digital solutions by youth is increasing worldwide, providing cost-effective opportunities for reaching this population with the delivery of self-help educational programs. Also, recent studies support our findings, showing that digital interventions based on chatbots or videogames can be highly engaging, improve well-being, and reduce stress for this population and for nonclinical populations [19-21].

The chatbot intervention that was co-designed and evaluated in this study was rather easy and fast to implement and was well-received by adolescents. The two phases of our participatory design process took into account the feedback and suggestions of an overall sample of 41 adolescents; the results reported can guide the future development of virtual coaching solutions for adolescents’ training, solutions that have been found to be acceptable and appealing to use for this target user group. The deployment of educational video cartoons and dialogue-based interaction with the chatbot turned out to be very engaging and useful for self-reflecting on the challenging situations presented, making the virtual coaching sessions a very promising digital environment for experiential forms of learning in youth. The virtual coaching experience that was designed could easily be integrated into existing school programs and interventions for bullying and cyberbullying prevention; this coaching experience could also be considered a practical way of providing out-of-school interventions for life skills training.

This study is limited by the participant sample size and by having been conducted only in Northeast Italy, which impacts the generalizability of our results to other countries and settings. However, mental health promotion programs for adolescents provided by a variety of delivery platforms, including digital media, are strongly recommended by public health policies in many countries and by the World Health Organization [22]. These recommendations support the international relevance of our study.

### Conclusions

In summary, this study presented the co-design and formative evaluation of a chatbot-based coaching intervention for adolescents’ life skills training, which was grounded in a positive technology approach and was well-received by adolescents. Further research is needed for a more in-depth evaluation of the efficacy of this intervention in strengthening coping strategies and resilience.

A future step of this study consists of integrating our chatbot intervention on life skills into the Cyberbullying Effects Prevention (CREEP) platform for cyberbullying prevention, which will be tested for efficacy by involving approximately 200 secondary school students in Italy and France.

## Appendix

Multimedia Appendix 1 Video of the chatbot-user interaction for the coaching session on conflict resolution.

## Abbreviations

CREEP: Cyberbullying Effects Prevention
EIT: European Institute of Innovation and Technology

## References

[1] Giedd JN (2008). The teen brain: Insights from neuroimaging. J Adolesc Health.

[2] Patchin JW (2019). Cyberbullying Research Center.

[3] Craig W, Harel-Fisch Y, Fogel-Grinvald H, Dostaler S, Hetland J, Simons-Morton B, Molcho M, de Mato MG, Overpeck M, Due P, Pickett W, HBSC Violence & Injuries Prevention Focus Group, HBSC Bullying Writing Group (2009). A cross-national profile of bullying and victimization among adolescents in 40 countries. Int J Public Health.

[4] Nakamoto J, Schwartz D (2010). Is peer victimization associated with academic achievement? A meta-analytic review. Soc Dev.

[5] Hawker DS, Boulton MJ (2000). Twenty years' research on peer victimization and psychosocial maladjustment: A meta-analytic review of cross-sectional studies. J Child Psychol Psychiatry.

[6] Copeland WE, Wolke D, Angold A, Costello EJ (2013). Adult psychiatric outcomes of bullying and being bullied by peers in childhood and adolescence. JAMA Psychiatry.

[7] Botvin G (2000). J. Life Skills Training.

[8] Birrell Weisen R, Orley J, Evans V, Lee J, Sprunger B, Pellaux D, World Health Organization. Division of Mental Health (1997). Life Skills Education for Children and Adolescents in Schools. Pt. 1, Introduction to Life Skills for Psychosocial Competence. Pt. 2, Guidelines to Facilitate the Development and Implementation of Life Skills Programmes, 2nd revision.

[9] Srikala B, Kishore KK (2010). Empowering adolescents with life skills education in schools - School mental health program: Does it work?. Indian J Psychiatry.

[10] Botella C, Riva G, Gaggioli A, Wiederhold BK, Alcaniz M, Banos RM (2012). The present and future of positive technologies. Cyberpsychol Behav Soc Netw.

[11] Riva G, Baños RM, Botella C, Wiederhold BK, Gaggioli A (2012). Positive technology: Using interactive technologies to promote positive functioning. Cyberpsychol Behav Soc Netw.

[12] Lenhart A (2015). Pew Research Center.

[13] Free C, Phillips G, Watson L, Galli L, Felix L, Edwards P, Patel V, Haines A (2013). The effectiveness of mobile-health technologies to improve health care service delivery processes: A systematic review and meta-analysis. PLoS Med.

[14] Fitzpatrick KK, Darcy A, Vierhile M (2017). Delivering cognitive behavior therapy to young adults with symptoms of depression and anxiety using a fully automated conversational agent (Woebot): A randomized controlled trial. JMIR Ment Health.

[15] Pereira J, Díaz Ó (2019). Using health chatbots for behavior change: A mapping study. J Med Syst.

[16] Braun V, Clarke V (2006). Using thematic analysis in psychology. Qual Res Psychol.

[17] Faulkner L (2003). Beyond the five-user assumption: Benefits of increased sample sizes in usability testing. Behav Res Methods Instrum Comput.

[18] Boddy CR (2016). Sample size for qualitative research. Qual Mark Res.

[19] Ly KH, Ly A, Andersson G (2017). A fully automated conversational agent for promoting mental well-being: A pilot RCT using mixed methods. Internet Interv.

[20] Carissoli C, Villani D (2019). Can videogames be used to promote emotional intelligence in teenagers? Results from EmotivaMente, a school program. Games Health J.

[21] Young Oh E, Song D, Hong H (2019). Interactive computing technology in anti-bullying education: The effects of conversation-bot’s role on K-12 students’ attitude change toward bullying problems. J Educ Comput Res.

[22] (2019). World Health Organization.

